# Supplementary material for: A Humanized Yeast Phenomic Model of Deoxycytidine Kinase to Predict Genetic Buffering of Nucleoside Analog Cytotoxicity
Source: Genes (Basel). 2019 Sep 30;10(10):770. doi: 10.3390/genes10100770 (PMC6826991; doi:10.3390/genes10100770)
Supplement: Supplementary file 1 [file genes-10-00770-s001.zip › Additional_Files/Additional_File7_GOTermHeatmaps.zip!/Additional_File7_GOTermHeatmaps/transcription_coactivator_activity.pdf]

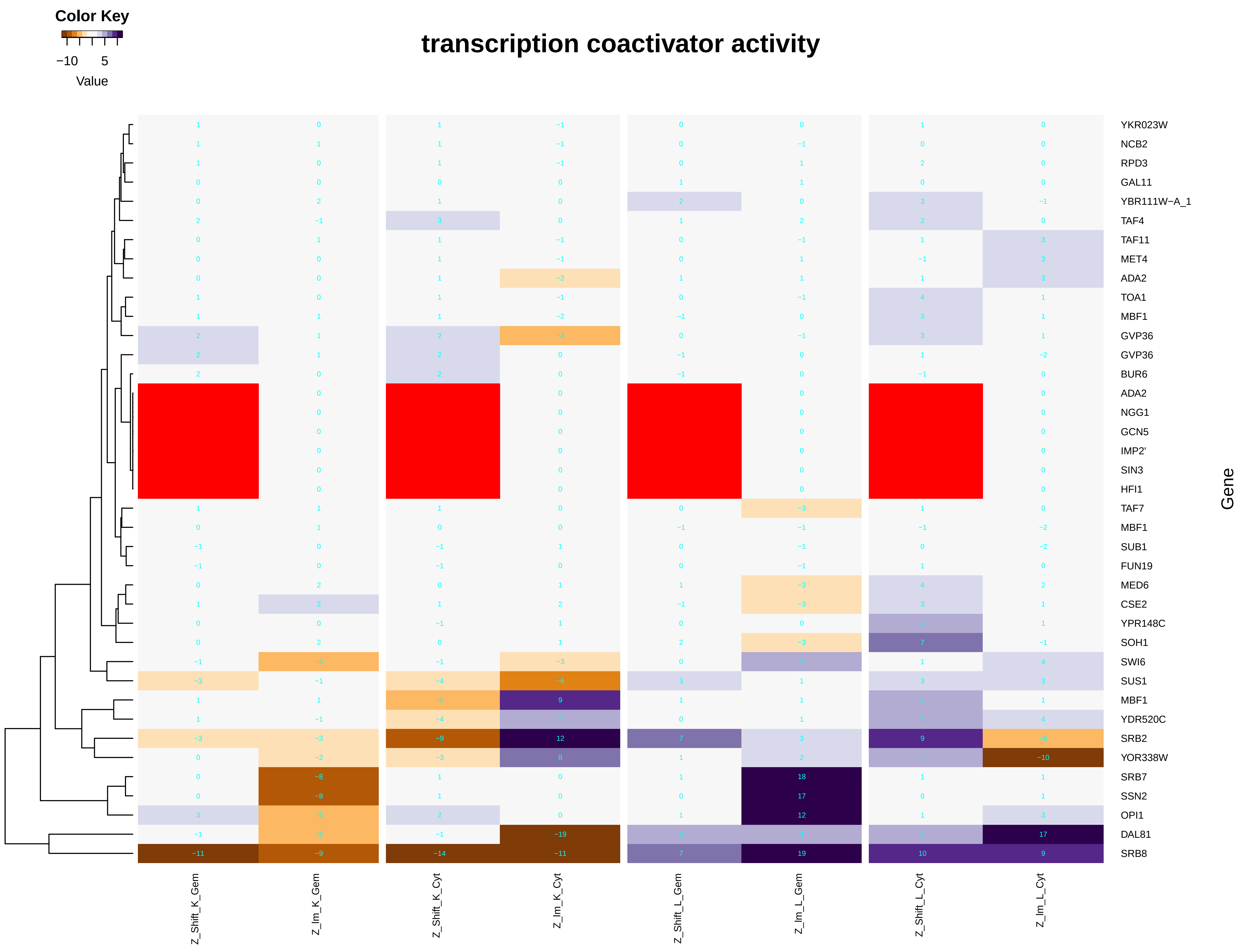

transcription coactivator activity
Color Key
−10 5
Value
Gene
YKR023W
NCB2
RPD3
GAL11
YBR111W−A_1
TAF4
TAF11
MET4
ADA2
TOA1
MBF1
GVP36
GVP36
BUR6
ADA2
NGG1
GCN5
IMP2'
SIN3
HFI1
TAF7
MBF1
SUB1
FUN19
MED6
CSE2
YPR148C
SOH1
SWI6
SUS1
MBF1
YDR520C
SRB2
YOR338W
SRB7
SSN2
OPI1
DAL81
SRB8
Z_Shift_K_Gem
Z_Im_K_Gem
Z_Shift_K_Cyt
Z_Im_K_Cyt
Z_Shift_L_Gem
Z_Im_L_Gem
Z_Shift_L_Cyt
Z_Im_L_Cyt

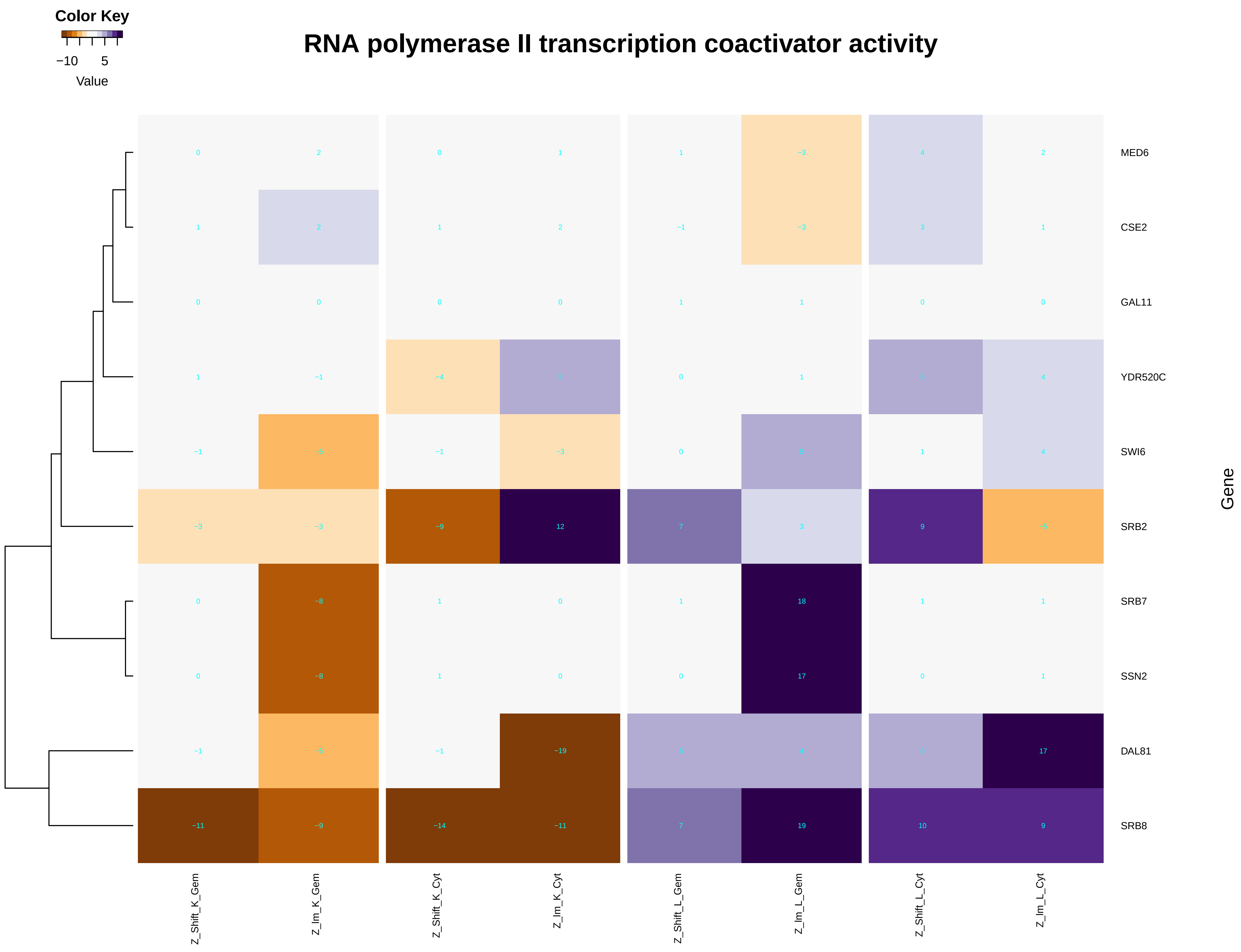

RNA polymerase II transcription coactivator activity
Color Key
−10 5
Value
MED6
CSE2
GAL11
YDR520C
SWI6
SRB2
SRB7
SSN2
DAL81
SRB8
Gene
Z_Shift_K_Gem
Z_Im_K_Gem
Z_Shift_K_Cyt
Z_Im_K_Cyt
Z_Shift_L_Gem
Z_Im_L_Gem
Z_Shift_L_Cyt
Z_Im_L_Cyt

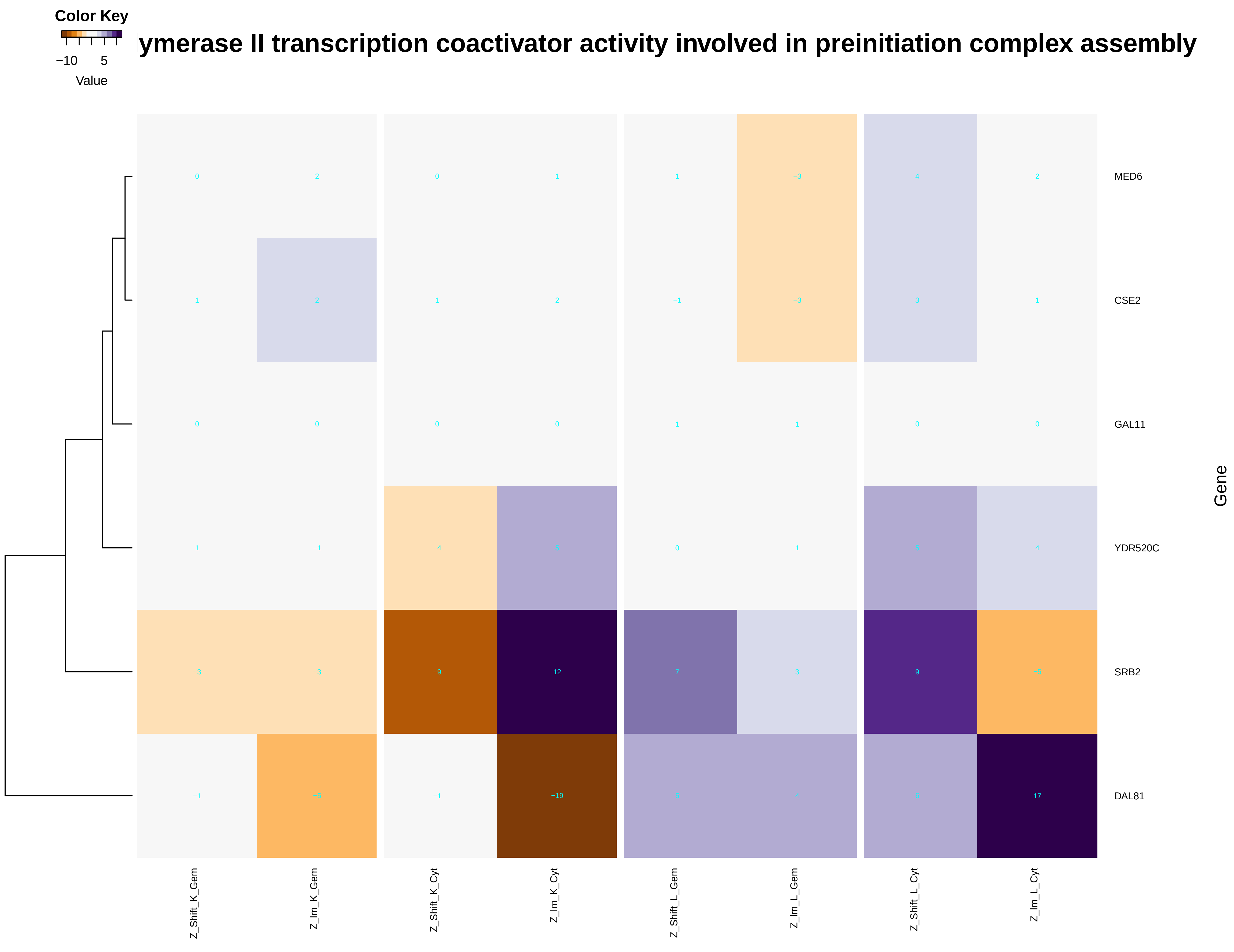
Color Key
−10
5
Value
ymerase II transcription coactivator activity involved in preinitiation complex assembly
MED6
CSE2
GAL11
YDR520C
SRB2
DAL81
Gene
Z_Shift_K_Gem
Z_lm_K_Gem
Z_Shift_K_Cyt
Z_lm_K_Cyt
Z_Shift_L_Gem
Z_lm_L_Gem
Z_Shift_L_Cyt
Z_lm_L_Cyt
